# Specific or general exercise strategy for subacromial impingement syndrome–does it matter? A systematic literature review and meta analysis

**DOI:** 10.1186/s12891-017-1518-0

**Published:** 2017-04-17

**Authors:** Alison R. Shire, Thor A. B. Stæhr, Jesper B. Overby, Mathias Bastholm Dahl, Julie Sandell Jacobsen, David Høyrup Christiansen

**Affiliations:** 1 Department of Physiotherapy, VIA University College, Hedeager 2, Aarhus N, Denmark; 2 Danish Ramazzini Centre, Department of Occupational Medicine, University Research Clinic, Regional Hospital West Jutland, Herning, Denmark

**Keywords:** Impingement, Subacromial, Shoulder, Physiotherapy, Training, Function, Pain

## Abstract

**Background:**

Exercise is frequently suggested as a treatment option for patients presenting with symptoms of subacromial impingement syndrome. Some would argue implementing a specific exercise strategy with special focus on correction of kinematic deficits would be superior to general exercise strategy. There is however a lack of evidence comparing such exercise strategies to determine which is the most effective in the treatment of subacromial impingement syndrome. The aim of this review is to evaluate whether implementing specific exercise strategies involving resistive exercises are more effective than a general exercise strategy for the treatment of patients with subacromial impingement syndrome.

**Methods:**

Randomized controlled trials were identified through an electronic search on PubMed/MEDLINE, EMBASE, Cochrane Central Register of Controlled Trials, Web of Science and PEDro. In addition, article reference lists and Clinicaltrials.gov were searched. Studies were considered eligible if they included interventions with resistive specific exercises as compared to general resistance exercise. Four reviewers assessed risk of bias and methodological quality guided by Cochrane recommendations. Results were synthesised qualitatively or quantitatively, where appropriate.

**Results:**

Six randomized controlled trials were included with 231 participants who experienced symptoms of subacromial impingement syndrome. Four studies evaluated the effectiveness of specific scapular exercise strategy and two studies evaluated the effectiveness of specific proprioceptive strategy. Five studies were of moderate quality and one study was of low quality. No consistent statistical significant differences in outcomes between treatment groups were reported in the studies. Standardized mean difference (SMD) for pain was SMD −0.19 (95% CI −0.61, 0.22) and SMD 0.30 (95% CI −0.16, 0.76) for function.

**Conclusions:**

There is insufficient evidence to support or refute the effectiveness of specific resistive exercise strategies in the rehabilitation of subacromial impingement syndrome. More high quality research is needed to accurately assess this. This review provides suggestions on how to improve the methodological design of future studies in this area.

**Electronic supplementary material:**

The online version of this article (doi:10.1186/s12891-017-1518-0) contains supplementary material, which is available to authorized users.

## Background

Subacromial Impingement Syndrome (SIS) is thought to be the most prevalent disorder causing shoulder pain, accounting for up to 50–70% of all shoulder complaints in primary care [1–3]. The term shoulder impingement syndrome was introduced by Neer to describe the pathological state of the rotator cuff tendons resulting from mechanical impingement [4, 5].

Subacromial impingement syndrome is a multifactorial condition where intrinsic and extrinsic mechanisms of rotator cuff pathology are the two main theories underlying SIS aetiology [6, 7]. Intrinsic factors affect tendon morphology and performance over time. These factors are influenced by genetic predisposition, age related changes, poor vascularity, biological alterations and mechanical properties where the physiological limit of the rotator cuff tendon(s) are surpassed [6–8]. Extrinsic factors relate to anatomical structure and/or biomechanical alterations causing mechanical compression [5, 7]. Anatomical structures include variations of the acromion shape as well as osteoarthritic spurs on the subacromial and acromioclavicular joints. Biomechanical alterations refers to the superior translation of humeral head and altered scapulohumeral kinematics often caused by a weakness and imbalance of the rotator cuff musculature and/or tendons opposing superiorly directed shifts as well as postural dysfunction [5, 8–13]. This can present as external impingement with narrowing of the subacromial space or internal impingement within the glenohumeral joint space [6, 8, 13].

Alterations in shoulder kinematics are often observed among patients with SIS [14–20]. The most common documented deficits are alterations in scapulothoracic kinematics [14, 16, 17, 20–26], humeral head displacement within the glenoid cavity [14, 16, 27, 28] and increased elevation and retraction of the clavicle in the sternoclavicular joint during arm elevation [14, 21, 24, 29]. Postural dysfunction relating to increased flexion and kyphosis of the thoracic spine cause alignment impairments which are also said to interfere with shoulder kinematics [16, 21]. These kinematic alterations are suggested to contribute to narrowing of the subacromial space (external impingement) which can affect biceps tendons, rotator cuff tendons, subacromial bursa and subtendinous bursa increasing the risk of joint inflammation and tears associated with SIS [9, 14–16, 19]. Physiotherapists often tailor rehabilitation programs to correct movement deficits, postural dysfunction and or muscles weakness/imbalance in attempted to improve characteristics of the subacromial space [16, 19, 21].

Exercise therapy has shown to reduce pain and improve functional loss associated with SIS, however specific components of exercise protocols are unknown [18, 30–34]. A systematic review from 2012 concluded that a program consisting of multiple types of exercises are effective in the rehabilitation of SIS [32]. These programs consist of a combination of scapular stabilization exercises, rotator cuff resistance exercises, range of motion and stretching exercises. Current evidence is however limited as to the which specific exercise(s) are most effective clinically [32, 35]. However, there is growing evidence to support the use of resistance and proprioceptive exercises over movement based exercises alone [18, 28, 32, 36]. A recent consensus statement from 2013 recommended that scapular kinematic deficits should be addressed with specific exercises in the rehabilitation of SIS [17]. This recommendation included specific exercises strategies aimed to restore normal scapular kinematics by improving the muscle activity, strength, flexibility and balance in muscle force couples that control scapular position and motion [14, 17, 37–39]. In addition, Diedrichsen et al. [9] suggested that focus on motion awareness and strengthening of the scapular upward-rotators and the rotator cuff should be part of a conservative rehabilitation program for patients with SIS.

An algorithm guideline on rehabilitation of shoulder injuries has been developed [18]. Recommendation for specific rotator cuff and scapular retraining includes muscle activation sequencing, force couple activation, concentric and eccentric control, strength, endurance and avoidance of unwanted movement patterns [18, 40, 41]. Key principles include obtaining flexibility in the muscles to reduce inhibition of activation and execution of specific functional movement or activity [18, 40, 41]. These specific strategies are thought to improve scapular kinematics and thereby ameliorate biomechanical conditions in the shoulder that may reduce symptoms caused by SIS [18, 40, 41]. Despite compelling research on exercise therapy and previous systematic reviews of the effectiveness of different exercise interventions, recently published trials justify an updated systematic review on the effectiveness of specific exercise strategies such as stabilization, positioning, proprioception, neuromuscular control, strengthening, stretching and centering of the humeral head. The aim of this review is to evaluate the outcome differences in treatment of SIS when comparing resistance training programs with and without specific exercise strategy.

## Methods

The reserach design and methodology for this literature review was based on recommendations from the Cochrane Handbook for Systematic Reviews of Interventions developed by the Cochrane Collaboration [42].

### Criteria for considering studies for this review

#### Types of studies

Randomized controlled trials (RCTs), published in all languages, investigating any approach of specific exercises in patients with SIS were considered.

#### Types of participants

Studies were included if participants were older than 18 years and demonstrated the clinical pattern of SIS. Studies were included that, according to examination, had participants presenting with at least one of the following signs of SIS: Pain with overhead activities; painful arc sign; positive Neer impingement test, Hawkins test or Jobes test.

Trials were excluded if they recruited participants with severe injuries including full thickness rotator cuff tears adhesive capsulitis (frozen shoulder), osteoarthritis, fractures/dislocations, neoplasm, systemic inflammatory and autoimmune disorders; infection, neurologic disorders or pain relating to complex neck/shoulder disorders. Postoperative rehabilitation interventions were not considered.

#### Types of interventions

To be considered for inclusion trials must include resistance exercises in both intervention and control group, and one of the groups must investigate the effect of a specific exercise strategy. Specific exercise strategies can be defined as exercise targeting the activation and coordination of scapulothoracic musculature and/or the dynamic humeral stabilizers that encompass the shoulder joint. Exercise can include scapular stabilization, positioning, proprioception, neuromuscular control, strengthening and stretching. Exercises must involve a form of resistance such as; body weight, elastic resistance, weighted apparatus, weights and/or machine weights.

#### Types of outcome measures

All outcomes were of interest. The primary outcomes considered were pain and function measured using shoulder-specific scales e.g. Western Ontario Rotator Cuff Questionnaire (WORC) and Shoulder Disability Questionnaire (SDQ). Quality of life (QoL), clinical tests, range of motion (ROM), strength and proprioceptive ability were considered as secondary outcomes.

### Search methods for identification of studies

Two review authors conducted an electronic search of

1. PubMed (MEDLINE)
2. EMBASE
3. Cochrane Central Register of Controlled Trials (CENTRAL)
4. Physiotherapy Evidence Database (PEDro)
5. Web of Science

The search strategy was constructed after consultation with an academic health science librarian. The following index and key free-text terms were used: shoulder, scapula, subacromial, impingement, bursitis, tendinitis, tendonitis, rehabilitation, physiotherapy, physical therapy, exercise and training. All key terms were searched independently and then combined [42]. The search formulas for each database are provided in Appendix 1. Reports not indexed in the databases were manually searched and detected. Furthermore, retrieved articles and systematic reviews were screened for additional relevant publications. A search in clinicaltrials.gov of recent listed studies/unpublished articles was also performed.

### Data collection and analysis

#### Selection of studies

Two review authors (TS, AS) independently applied the inclusion and exclusion criteria to screen the titles and abstracts. Secondly, reviewers (TS, AS) retrieved the potential eligible studies in full-text and evaluated the aim and methods sections for final inclusion. Two other reviewers (JO, MD) compared article selection and resolved any disagreements by consensus. If disagreements persisted, another review author was consulted (DC). All six reviews (TS, AS, JO, MD, DC and JJ) read the eligible studies in full-text and came to an agreement on the six included studies.

#### Data extraction and management

Two pairs of review authors (AS, MD and TS, JO) independently extracted data from the included studies. Study characteristics regarding methods, participants, interventions, outcomes and results were extracted using standardized data extraction forms in preparation for accurate analysis [43]. Original paper authors were contacted to obtain more information if needed.

#### Risk of bias assessment

Risk of bias in the included studies was assessed by two independently blinded groups of review authors (AS, MD and TS, JO) and then compared and discussed by all four reviewers.

Any disagreements were resolved by consulting a fifth review author (DC). The level of inter-rater agreement was recorded. Data extraction and evaluation of risk of bias was conducted using Review Manager 5.3 [44].

Risk of bias was assessed by utilizing the 12 criteria adapted by Cochrane from van Tulder et al. [45], Boutron et al. [46] and Furlan et al. [43] The criteria were used to validate characteristics of the studies that might be related to reporting, selection, performance, attrition and detection bias [42]. A pilot risk of bias assessments was performed with similar articles to ensure consistent interpretation of the criteria between reviewers.

In addition to the risk of bias assessment the reviewers investigated other methodological considerations even though it was not stated in the protocol e.g. sample size and interim analyses. With reference to a Cochrane review, sample size was considered inadequate if less than 50 participants per group and/or if power analysis was not applied and reported for relevant outcome measures [47]. Groups should be adequately powered for detecting a 20% relative difference in the relevant outcome. In this process the review authors focused on six key domains perceived crucial for a study’s methodological quality: randomization, treatment allocation, intention-to-treat analysis, compliance, drop-out rate and in addition to this; other bias resulting in serious flaws. According to these key domains the authors determined whether each study had a high, unclear or low risk of bias (see Table 1). Given the non-pharmacologic nature of investigated interventions contributing to difficulties with blinding of care providers and patients, the review authors chose not to focus on blinding as a key domain [48]. Patients had knowledge of their intervention and outcome measures were patient reported. For example when assessing pain, patients act as outcome assessors thus patient blinding is impossible. Ethical issues such as the Helsinki declaration and consent create further difficulties for patient blinding [49].

**Table 1:** Going from assessments of risk of bias to judgments about study limitations

Risk of bias	Across studies	Interpretations	Considerations	GRADE assessment
Low risk of bias.	Most information is from studies at low risk of bias.	Plausible bias unlikely to seriously alter the results.	No apparent limitations.	No serious limitations, do not downgrade.
Unclear risk of bias.	Most information is from studies at low or unclear risk of bias.	Plausible bias that raises some doubt about the results.	Potential limitations are unlikely to lower confidence in the estimate of effect.	No serious limitations, do not downgrade.
			Potential limitations are likely to lower confidence in the estimate of effect.	Serious limitations, downgrade one level.
High risk of bias.	The proportion of information from studies at high risk of bias is sufficient to affect the interpretation of results.	Plausible bias that seriously weakens confidence in the results.	Crucial limitation for one criterion, or some limitations for multiple criteria, sufficient to lower confidence in the estimate of effect.	Serious limitations, downgrade one level
			Crucial limitation for one or more criteria sufficient to substantially lower confidence in the estimate of effect.	Very serious limitations, downgrade two levels.

Adapted from Table 12.2.d from Cochrane Handbook [42]. Further guidelines for factor 1 (of 5) in a GRADE assessment: Going from assessments of risk of bias to judgments about study limitations for main outcomes

#### Best evidence synthesis

The quality of each individual study was rated. Included RCTs were initially considered of high quality and then downgraded on the basis of the key domains (see Table 2).

**Table 2:** Levels of Quality

Underlying methodology	Quality rating
Randomized trials; or double-upgraded observational studies.	High
Downgraded randomized trials; or upgraded observational studies.	Moderate
Double-downgraded randomized trials; or observational studies.	Low
Triple-downgraded randomized trials; or downgraded observational studies; or case series/case reports.	Very low

Adopted from Cochrane Handbook [42]: Levels of quality of a body of evidence in the GRADE approach

The effectiveness and level of evidence for each outcome of interest across studies were evaluated by application of “best evidence synthesis guidelines” as presented in Dorrestijn et al. [50] modified from the one proposed by Van Tulder et al. [45] (see Table 3). This was used by the reviewers to determine whether each outcome was of strong, moderate, limited or no/insufficient evidence. The quality classification was then combined with evaluated evidence to determine the strength of evidence for each outcome.

**Table 3:** Best evidence synthesis guidelines

Strong evidence	Provided by consistent^a^ statistically significant findings in outcome measures in at least two high quality RCTs^b^
Moderate evidence	Provided by statistically significant findings in outcome measures in at least one high quality RCT^b^ or Provided by consistent^a^, statistically significant findings in outcome measures in at least two medium quality RCTs^b^
Limited evidence	Provided by statistically significant findings in at least one medium quality RCT^b^ or Provided by consistent^a^, statistically significant findings in outcome measures in at least two low quality RCTs^b^
No or insufficient evidence	If results of eligible studies do not meet the criteria for one of the levels of evidence listed above (e.g. no statistically significant findings) or In case of conflicting (statistically significant positive and statistically significant negative) results among RCTs or In case of no eligible studies

Best evidence synthesis guidelines as modified by Dorrestijn et a [50] from the synthesis by van Tulder et al. [45]

*Abbreviations*: *RCT* randomized controlled trial

^a^ Findings are considered consistent if they point in the same direction

^b^ If the number of studies showing evidence is lower than 50% of the total number of studies found within the same category of methodological quality, we state no evidence

### Data synthesis and analysis

Data analysis was conducted using Review Manager (version 5.3) [44] of the Cochrane Collaboration and consult of a statistician. Studies were included in the quantitative analysis if the primary outcomes of interest, pain and function, were descriptively comparable at baseline and follow-up in regards to intervention, participants, outcome measures and duration of follow-up. Where possible the means and standard deviations data pain and function of included studies were pooled into a meta-analysis to give the overall summary of effect. Data was converted and calculated to standardized mean difference (SMD) with 95% confidence intervals (CIs) for short term outcomes which ranged from 4 to 8 weeks.

A random effects model was used to determine the overall effect size [42, 51]. An effect size of 0.8 or more was regarded as a large effect size, between 0.5 and 0.8 as a moderate effect size and between 0.2 and 0.5 as a small effect size [52]. Statistical significance was considered at *p* < 0.05.

Forest plots were used to illustrate effect sizes on pain and function with 95% CIs and to summarize the pooled effect. Funnel plots to identify publication bias were not generated because of the small number of studies available for each analysis. For crossover trials only data from the first period were included [53]. The outcomes measures of pain during movement and function were found comparable across five studies [53–57]. The function scoring scales were reversed in two studies [55, 57] for the adequate interpretation and to enable meta-analysis of the data. To ease interpretation the function scores in two studies were converted to 0–100 [56, 57] and in one study the pain scores were converted to 0–10 [56].

The degree of heterogeneity was assessed by I^2^ statistics. The I^2^ can be interpreted as the proportion of the observed discrepancy in the estimation of the effect, within a group of trials, which cannot be accounted for by random variation [42]. Due to the low number of studies currently available the authors did not restrict the inclusion of studies with high risk of bias. Review authors performed the recommended sensitivity analyses in order to provide a transparent conclusion [42]. The methodological factors such as age, sex, intervention and the follow-up periods of each study were investigated to explore and explain the factors of heterogeneity.

## Results

### Selection of studies

**Fig. 1:**
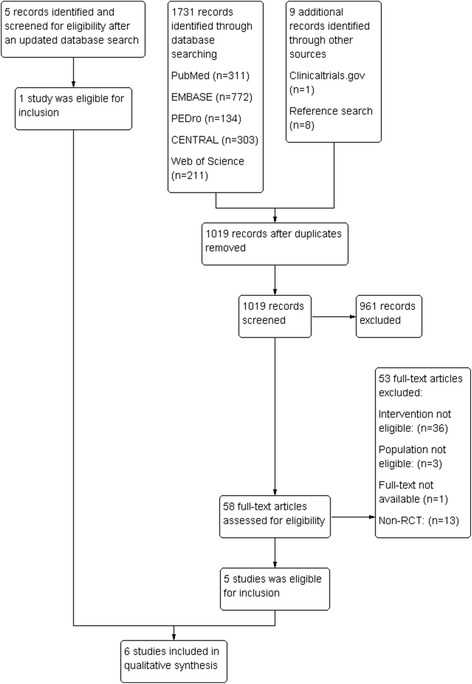
PRISMA flow chart of inclusion of studies. The flow of the search and selection process in this systematic review and meta-analysis of specific exercises for patients with subacromial impingement syndrome. Review Manager (RevMan) 5.3 [44, 71]

Databases were searched within a 2-day period retrospective of inception to September 2015 with a subsequent update to June 2016. The initial database search resulted in 1731 hits and the manual search in nine hits (including reference search), which reduced to 1019 after deletion of duplicates (Fig. 1). After screening titles and abstracts, 961 studies were deemed irrelevant and excluded; whereas 58 studies were eligible for full-text screening. The subsequent update yielded 1 eligible study (Fig. 1). Overall a total of six that were deemed fit for inclusion. Excluded studies are accounted for in Appendix 2 and characteristics of included studies are described in Table 4. One ongoing study was identified in clinicaltrials.gov (see Appendix 3).

**Table 4:** Characteristics and evaluation of included studies

Study	Method	Participants	Outcome measures	Results/comments	Key domains	Score	Quality
Baskurt 2011 [54]	Randomized by simple random table.	*n = 40, 13 male, 27 female.* *24*–*71 year. with a mean age of 51.*	Measured before and after intervention. Pain:VAS ROM: Goniometer Strength: Hand held dynamometer Function and QoL: WORC. Clinical tests: Joint Position Sense and Lateral Scapular Slide Test	Patients outcomes improved statistically in both groups (*P* < 0.05). No significant difference between groups in all parameters (*P* > 0.05) except muscle strength for lower trapezius and supraspinatus and clinical tests (*P* < 0.05). Comments: Missing *P*-values and CI in results section. No protocol registered.	Low risk: Randomization Drop-out rate Intention-to-treat Unclear risk: Allocation Compliance High risk: Other bias (sample size)	High risk (downgrade 1)	Moderate
Dilek 2016 [57]	Randomized using block randomization.	*n = 63, 21 male, 42 women.* *25*–*65 years. with a mean age of 49.13.* *Mean duration of symptoms: approx. 17 months.*	Measured at baseline, 6 and 12 weeks. Pain: VAS ROM: Goniometer Strength: Isokinetic dynamometer system (Cybex Norm) Function and QoL: WORC and ASES	Both groups improved significantly in ROM, pain scores, isometric strength in all angles, Sense of kinesthesia at 0° ER, ASES and WORC (*P* < 0.05). No significant difference was found between groups in any of the parameters (*P* > 0.05). Comments: No protocol registered.	Low risk: Randomization Allocation Drop-out rate Intention-to-treat Unclear risk: Compliance High risk: Other bias (sample size)	High risk (downgrade 1)	Moderate
Martins 2012 [58]	Unclear randomization	*n = 18, nursing professionals with age ranging from 30 to 50+ yrs.* *16 subjects completed the study consisting of 2 male and 14 females. (no information on gender n = 2)*	Pain: VNS Function and QoL: WORC	Both groups exhibited significant reduction in pain scores (*P* < 0.05), however, no significant differences between groups. Only the intervention group improved WORC scores significantly. Comments: Outcomes of pain was less well presented and reduces transparency of data.	Low risk: Allocation Drop-out rate Intention-to-treat Compliance Unclear risk: Randomization High risk: Other bias (sample size)	High risk (downgrade 1)	Moderate
Mulligan 2016 [53]	Randomized using blind draw.	*n = 50, 14 male and 26 females, (no information on gender n = 10)*	Measured at baseline, 4 weeks, 8 weeks, 16 weeks Pain: NPRS Function and QoL: ASES; GPF; GROC.	No significant between group and time. Both groups exhibited significant reduction in pain scores and function. However, no significant differences between groups. Comments: Protocol registered. Unclear reporting of co-interventions.	Low risk: Randomization Drop-out rate Intention-to-treat Allocation Unclear risk: Compliance High risk: Other bias (sample size)	High risk (downgrade 1)	Moderate
Struyf 2013 [55]	Randomized using closed envelopes.	*n = 22, 10 male and 12 females. Aging from 30 to 61 years with a mean age of 45.8.*	Measured at baseline, after nine sessions (4–8 weeks) and 12 weeks Post treatment. Pain:VAS and VNRS Function and QoL: SDQ Clinical measures: Strength: handheld Dynamometer, Impingement test VAS Hawkins, Empty Can or Neer tests. Acromial distance, pectoralis minor length, scapula upward rotation and kinetic medial rotation test.	After nine sessions the Experimental group demonstrated significant effect on self-reported disability compared to the Control group (*P* = 0.025). Both groups increased from baseline in all outcomes measured (*P* < 0.05). No significant differences between group for strength or clinical measures, with exception for VAS by Neers test (*p* = 0.02) Comments: The protocol states that outcomes will be measured after 6 months. It is reported interim analysis was planned, however, is not mentioned in the protocol.	Low risk: Randomization Allocation Intention-to-treat Drop-out rate Unclear risk: Compliance High risk: Other bias (sample size and interim analyze)	High risk (downgrade 1)	Moderate
Wang 2006 [56]	Randomized using pre-prepared sealed envelopes.	*n = 38, aging from 26 to 68 years. with a mean age of 44.6. Of the 38 subjects, 30 were analyzed consisting of 15 male and 15 female.*	Measured at baseline, 4 and 8 weeks. Pain: VAS Function and QoL: FLEX-SF ROM: Goniometer Strength: handheld dynamometer	No significant interaction between group and time. Both groups had significant improvements in regards to pain, function and muscle strength. FLEX-SF improved significantly after 8 weeks. Pain improved significantly after 4 and 8 weeks. Comments: No protocol registered. No gender distribution of the included subjects.	Low risk: Randomization Intention-to-treat Compliance Unclear risk: Allocation High risk: Drop-out rate Other bias (sample size)	High risk (downgrade 2)	Low

*Abbreviations*: *VAS* Visual Analog Scale, *ROM* Range of mortion, *QoL* Quality of life, *WORC* Western Ontario Rotator cuff Index, *CI* Confidence intervals, *ASES* American Shoulder and Elbow Surgeons Standardized Assessment Form-self reported scale, *GPF* Global Percentage of Function, *GROC* Global Rating of Change Scale, *ER* External rotation, *VNS* Verbal Numeric Scale, *SDQ* Shoulder Disability Questionnaire, *FLEX-SF* Flexilevel Scale of Shoulder Function, *VNRS* Verbal Numeric Rating Scale, *NPRS* Numeric Pain Rating Scale

### Description of studies

Of the six included studies a total of 231 participants were recruited [53–58]. Gender was reported for 211 participants, 136 women and 75 men; however, one study did not report gender for eight participants [56], another for two participants [58] and another for ten participants [53]. Four studies included patients with SIS and used similar clinical diagnosis criteria [53–55, 57]. One study included patients with SIS, but did not describe how diagnosis was made [58]. One study did not clearly address SIS, but rotator cuff disease [56].

The sample size of all studies was small ranging from 16 to 63 subjects [53–58]. Duration of symptoms at baseline were reported in five studies [53, 55–58], however, missing in one study [54].

Across the six studies the following outcomes were measured; Pain (at rest, during activity, worst pain during the last 24 h, during night), function and QoL (WORC, American Shoulder and Elbow Surgeons Standardized Assessment Form-self reported scale (ASES), SDQ, Flexilevel Scale of Shoulder Function (FLEX-SF)), strength and ROM [53–58]. Pain was measured using the following instruments; visual analogue scale (VAS), visual numeric scale (VNS), verbal numeric rating scale (VNRS) or Numeric Pain Rating Scale (NPRS) [53–58]. All studies reported function [53–58].

### Description of interventions

Two studies had similar intervention groups focusing on specific proprioceptive exercises and centering of the humeral head (positioning) [57, 58], one study compared scapular specific exercises for the shoulder against general resistance exercises [55] and two studies compared general resistance exercises for the shoulder against the same program with the addition of specific scapular stabilization and neuromuscular control exercises [54, 56]. One study was designed as a crossover trial where each group performed the same specific resistance scapular stabilization and rotator cuff exercises in a different sequence with follow-up at 4, 8, 12 and 16 weeks [53]. For the purpose of this review this study was analyzed at 4 weeks/follow-up before execution of crossover sequence [53]. Two studies reported measurements after the intervention at 4 to 8 weeks [55] and 6 weeks follow-up [54]. One study reported on 4 and 8 weeks follow-up [56] and one study reported on 6 and 12 weeks follow-up [57]. The timeframe of follow-up was unclear in one study [58].

Interventions of all studies lasted between 4 and 8 weeks [53–58], with one study encouraging participants to continue exercise at home for 6 weeks after the intervention [57]. Frequency of interventions varied between trials: Three times per week [54, 57], twice per week [56], once a day [53, 55] and twice a day [58]. Exercise protocols included: scapular specific and neuromuscular exercises [53–58], strengthening exercises of the shoulder and rotator cuff using gravity, TheraBand™ or free weights as resistance [53, 54, 56–58], stretching and flexibility exercises [54–58], proprioceptive training [54, 57] and general movement exercises [54, 57, 58]. One study selected exercises based on reported high electomyographical activity in target muscle groups [53].

In summary, all six studies investigated relevant specific exercise strategies over a short term [53–58]; Four studies evaluated the effectiveness of specific scapular exercise strategy [53–56] and two evaluated the effectiveness of specific proprioceptive exercise strategy [55, 57].

### Risk of bias assessment and quality rating

**Fig. 2:**
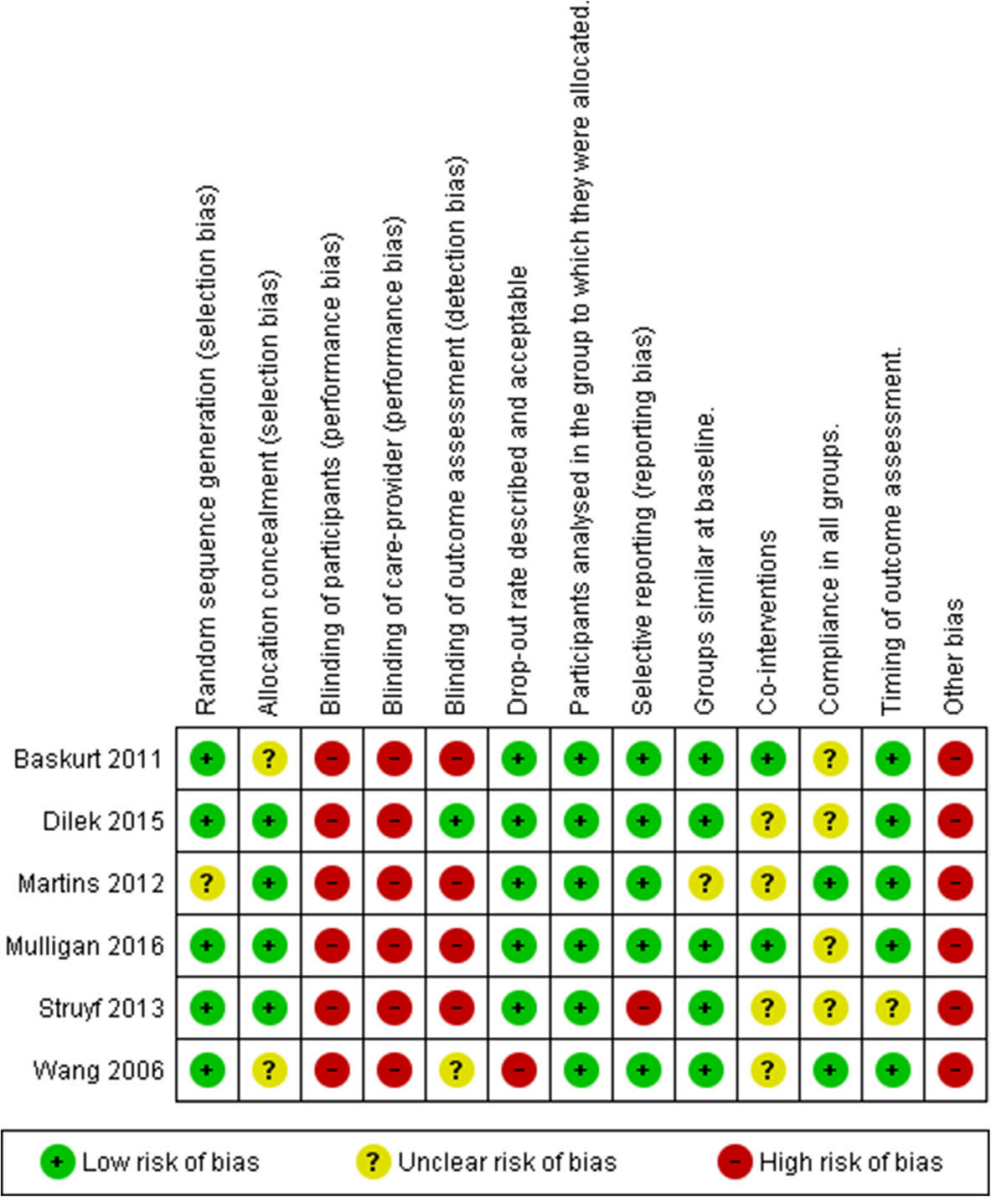
Risk of bias summary. This graph illustrates the review authors assessment of each risk of bias domain for the six included studies. Review Manager (RevMan) 5.3 [44]

**Fig. 3:**
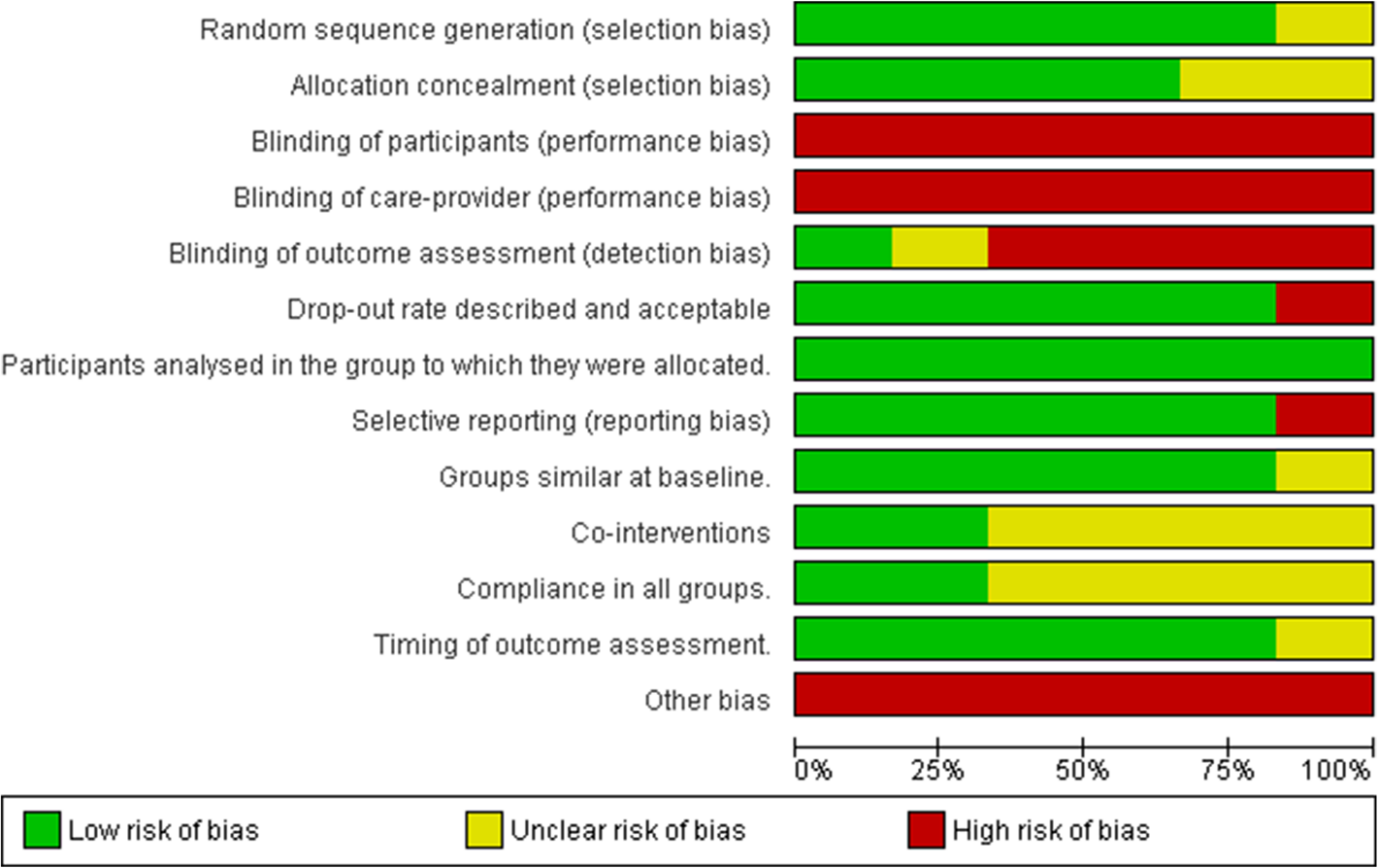
Risk of bias summary. This graph illustrates the review authors assessment of each risk of bias domain presented as percentages for the six included studies. Review Manager (RevMan) 5.3 [44]

The risk of bias in each study is presented in the last column in Table 4 and the overall risk of bias across studies is summarized in Figs. 2 and 3. Each study was evaluated on 12 risk of bias criteria equivalent to a total of 72 across included studies. The review groups agreed on 55 out of the 72 criteria. Final evaluation for eight risk of bias criteria were resolved by consensus and the remaining nine criteria were resolved by consulting a fifth review author. Review groups agreed on all parameters regarding other bias.

The most common methodological deficiency was small sample size. Randomization was performed in all studies. Five studies described random allocation appropriately [53–57]; whereas one study had unclear randomization methods [58]. Another prevalent methodological downfall is the lack of allocation concealment, which was unclear in three out of six studies [53, 54, 56]. The drop-out rate was considered higher than acceptable in one study [56]. All studies analyzed participants by intention to treat principle [53–58]. Selective outcome reporting was of high risk in one study [55]. One study scored unclear for similarity of groups at baseline [58]. Co-interventions were utilized in all studies, but only two were low risk of bias [54, 55]. Four studies failed to report compliance [53–55, 57]. One study failed to address timing of outcome assessment appropriately [55]. This study also failed to specify stopping rules for the performance of their interim analysis contributing to a high risk of bias and a downgrade in evidence quality [55]. All studies had inadequate sample sizes [53–58]. In summary, all studies were deemed to have a high risk of bias [53–58].

### Best evidence synthesis

Five studies were deemed to be of moderate quality [53–55, 57, 58] and one of low quality [56]. Specific exercises were employed by all six studies [53–58]. The results for overall effectiveness of specific exercise strategy on pain, function, strength, ROM proprioception and other clinical test were analyzed with best synthesis across outcomes are summarized in Table 5.

**Table 5:** Overall effectiveness of specific exercise strategy and best evidence synthesis across outcomes on short term

Outcome	Inter-group effectiveness^a^	Best evidence synthesis
Pain	Yes (moderate [55]) No (moderate [53, 54, 57, 58], low [56])	Insufficient (conflicting) evidence
Function	Yes (moderate [55]) No (moderate [53, 54, 57, 58], low [56])	Insufficient (conflicting) evidence
General strength	Yes (moderate [54]) No (moderate [55, 57], low [56])	Insufficient (conflicting) evidence
^a^Scapular-stabilizers	Yes (moderate [54]) No (low [56])	Limited evidence
^a^Proprioception and clinical tests	No (moderate [55, 57]) Yes (moderate [54])	Insufficient evidence
Range of motion	No (moderate [54, 55, 57], low [56])	No evidence

^a^ Statistical significant effect between groups in favor of specific exercise intervention

#### Pain

All studies investigated the short-term effects of exercise on pain at 4 to 8 weeks follow-up using a range the measurement scales; VAS, VNS, VNRS or NPRS [53–58]. Pain was measured with one or in combination of the following: At rest, at night, with activity, during the last 24 h and/or with function. There was insufficient evidence to support that specific exercise strategies are effective in reducing pain short term. This is consistent with no statistically significant differences between groups in four studies of moderate quality [53, 54, 57, 58] and one study of low quality [56]. In contrast, only one moderate quality study reported a reduction in pain between groups with significant statistical difference [55].

#### Function

All studies investigated function of the shoulder using questionnaires to assess short-term symptoms, activities of daily living and QoL experienced among participants with SIS [53–58]. There is insufficient evidence that tailoring a specific exercise strategy can improve function. Three studies of moderate quality utilized WORC, where none of these studies found improvements between groups [54, 57, 58]. Two studies of moderate quality evaluated function using the ASES index and found no effect between groups [53, 57]. One study of low quality evaluated functional status using FLEX-SF and found no difference between groups [56]. The results of one study of moderate quality contradict the results of all other studies by finding a statistical significant improvement between groups when evaluating functional disability status using SDQ [55].

#### Strength

Four out of six studies investigated isometric strength of the shoulder using hand held dynamometer with different approaches [53–56]; whereas one study used isokinetic dynamometer system [57]. Only one study of moderate quality found a statistical significant difference between groups [54]. Two other studies of moderate quality [55, 57] and one of low quality [56] found no statistical significant difference. One study only measured muscular strength at baseline [53].

One study of moderate quality found that implementing specific exercises that focus on scapular stabilization were effective to improve scapular muscles strength with statistical significance between groups [54]. One study of low quality found no difference [56]. Overall, there is limited evidence to support the use of specific exercises to improve scapular muscles strength.

#### Proprioception and clinical tests

There is conflicting evidence that a specific exercise strategy can improve the clinical outcome measures of proprioceptive ability and shoulder function. Only one study of moderate quality investigated the effectiveness of specific proprioception exercises [57]. Another study of moderate quality implemented specific proprioceptive exercises in their rehabilitation program, however, did not address outcome measures and specify findings between the groups [58]. Two studies of moderate quality focusing on scapular specific exercises provided inconsistent results [54, 55]. These studies were unable to demonstrate an improvement for the clinical outcome measures such as pain provocation test, acromial distance, pectoralis minor muscle length, joint position sense and scapula scapular position and motion [54, 55].

#### Range of motion

There is no evidence a specific exercise strategy can improve ROM. This is due to insignificant statistical findings between the control and experimental groups in three studies of moderate quality [54, 55, 57], one of which only measured scapula motion and not general shoulder ROM [55], and one study of low quality [56]. Two studies of moderate quality did not address outcome measures of ROM [53, 58].

### Quantitative analysis

Four of the six studies were eligible for inclusion in the statistical pooling for pain [40, 53, 54, 56] and five of the six studies were eligible for function [53–57]. The pooling of data for strength, ROM and clinical tests were deemed inappropriate due to the considerable variation between measurement tools and outcomes used by all the studies. Attempts to contact authors to provide further data were unsuccessful. Overall, the studies included in the meta-analysis were few in number and the true effect sizes varied between studies.

### Pain

One study measured worst pain in the last 24 h [56] and one study measured pain during active shoulder elevation [53]. These measurements were assumed to be pain during movement and therefore included for statistical analysis.

**Fig. 4:**
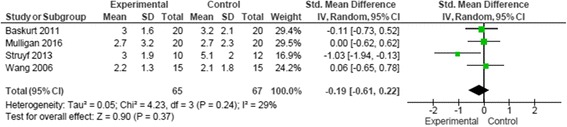
Data and forest plot illustrating results of specific exercise versus general exercise for short term pain during activity (4–8 weeks) [53–56]

Pooling of statistical data demonstrated no significant effect of specific exercise strategy on pain illustrated in Fig. 4 (SMD–0.19 (95% CI −0.61, 0.22)). Heterogeneity among the four included for pain outcomes [53–56] can be interpreted as not important (I^2^ = 29%) [42]. The sensitivity analysis revealed that heterogeneity for pain outcomes greatly affected by one study [55]. When this study [55] was removed from the analysis the degree of heterogeneity improved (I^2^ = 0%) and the effect size was reduced illustrated in Additional file 1: Figure S1.

### Function

One study measured function with ASES and WORC [57], but only means and standard deviations were reported for the WORC. One study [58] was excluded for both outcomes as pain was categorized without statistical data and data for function was not provided at baseline and follow-up.

**Fig. 5:**
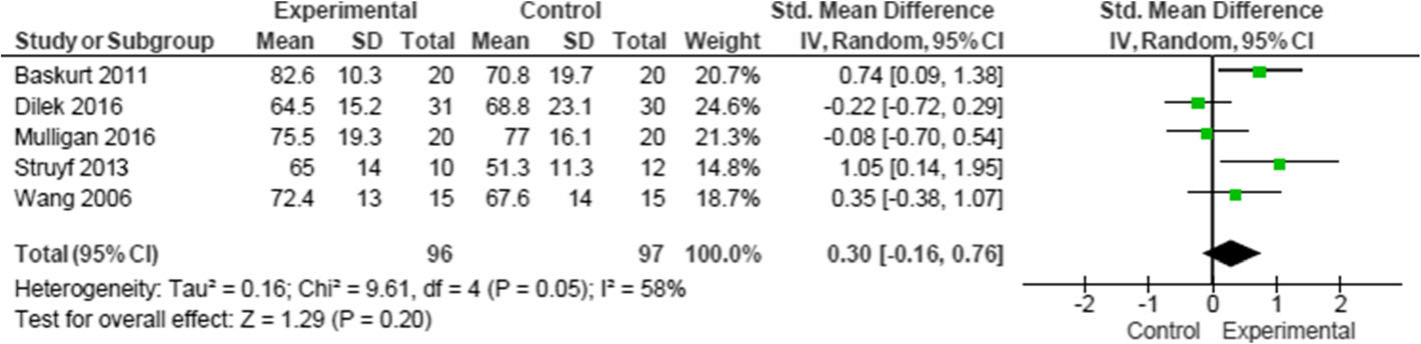
Data and forest plot illustrating results of specific exercise versus general exercise for short term function (4–8 weeks) [53–57]

Pooling of statistical data demonstrated no significant effect of specific exercise strategy on function at 4 to 8 weeks follow-up and are illustrated in Fig. 5 (SMD 0.30 (95% CI −0.16, 0.76)). Only one study analyzed function for a follow-up period of 12 weeks [57], however this did not influence the results with a repeat analysis of the data for 4 to 12 weeks follow-up.

Heterogeneity among the five studies for function outcomes [53–57] can be interpreted as moderate [42]. The amalgamation of the studies into different combinations of three studies [53, 56, 57] and [54–56] resulted in improved heterogeneity (I^2^ = 0%) illustrated in Additional file 1: Figure S1. The results from the combination of three studies did not favour the use of a specific exercise strategy [53, 56, 57]. In contrast, a moderate effect size was found in favour of specific exercise, when restricting the analysis to three other studies [54–56]. Consequently, the sensitivity analysis for function outcomes provide an unclear conclusion illustrated in Additional files 2 and 3: Figures S2 and S3.

## Discussion

The aim of this review was to evaluate whether implementing specific exercise strategy focused towards the treatment of SIS would result in a superior effect when compared to general exercises strategy in a resistance training program.

Six RCTs were extracted from a systematic search. Five of the studies were of moderate quality [53–55, 57, 58] and one of low quality [56]. All studies implemented a resistive specific exercises strategy in the form of proprioceptive, rotator cuff, scapular and stretching in different combinations. The qualitative results of this review suggest no significant evidence to support the use of specific exercises over general exercises in rehabilitation programs aiming to be an effective treatment for pain, function, ROM and strength in patients with SIS symptoms.

The most common cause for downgrading quality was the lack of adequate sample size which is found in all analyzed studies [53–58]. This increases the probability of inadequate equal distribution of participants with randomization, lack of power, and risk for statistical error [48]. Therefore, the ability to detect statistical significance and derive clinical meaning is reduced lowering overall confidence in the results of all studies [48].

Limitations such as poor intervention design and description limit transferability of protocols clinically. Unclear randomization [58] and allocation concealment [54, 56] are both methodological problems that introduce selection bias [42]. Unclear description and dosage of exercise were especially apparent in two of the studies [54, 56]. and in four studies it was unclear whether participants engaged in co-interventions [55–58].

One study had a relatively high drop-out rate which could underestimated efficacy of the intervention [56]. In contrast, applying simple imputation methods such a last observation carried forward, as used in one study [55], may introduce bias [48]. This could overestimate the efficacy of the intervention, especially if patients only are lost to follow-up in the comparative group. The compliance of participants was unclear in three studies [54, 55, 57], which create uncertainty as to whether the full intervention was received. Compliance is an important component of exercise as a treatment; therefore unclear reporting of compliance can make it difficult to determine true effectiveness. Non-compliance in physiotherapy interventions is as high as 70% [59]. Education for the rationale and perseverance of an exercise programs may be beneficial for both patient compliance and outcome measures [59].

The crossover design in one study employed the same intervention in both groups with different temporal sequence [53]. Regardless of the exercise type and sequence, both exercise strategies contributed to improvements in pain and function experienced by the subjects [53].

Most assessment tools utilized by included studies were valid and reliable for assessing outcomes measures among shoulder patients [53–58, 60, 61]. However, the reliability for strength, ROM and other clinical tests can be challenged and discussed. The variability of the clinical assessments/measures for function created methodological diversity among included studies. The tests used to assess strength, ROM and proprioception with scapular-specific tests in two studies [54, 55] are questionable as outcome measures for assessing the shoulder. There is a lack of validity and reliability which limit the clinical value of these tests [25, 60–62].

The results from the quantitative analysis for the primary outcomes of interest pain and function favour specific exercise strategies; however there was no significant statistical evidence proving them as a superior treatment.

Analyses of the heterogeneity among the studies revealed that inclusion and/or exclusion of one study [55] pulled results in different directions. For analysis of specific exercise strategies on pain the combination of three studies [53, 54, 56] resulted in zero heterogeneity and a small effect size not in favour of specific exercises. For function the combination of three studies resulted in zero heterogeneity and a moderate effect size [54–56]. It is difficult to pinpoint precisely which factors influence this variation of heterogeneity and as to why different combinations of studies result in a change of effect size. This reduces the clinical significance of the result. Disparity of clinical measures across all studies may be one factor contributing to the variation of heterogeneity. The deciding study [55] pulling results towards an effect of intervention presents methodological shortcomings when assessed using the Cochrane handbook and CONSORT criteria [42, 48]. Speculation of poor methodology and interventions cannot be dismissed and care should be taken when drawing conclusions estimating effect [42].

Attempts were made to improve the quality of evidence by pooling data from the eligible included studies for the quantitative analysis of pain and function. This however did not strengthen the results of this review and the conclusion remained affected.

Furthermore, all included studies were of high risk of bias and although this was addressed with sensitivity analysis strategies the inclusion of these studies in a meta-analysis lower the quality of evidence and power of this review [42]. Overall, this prevents a firm conclusion to support the use of specific exercises in clinical practice.

### Comparison with previous literature

Two previous systematic reviews investigated scapular-focused treatment strategies including; scapular-exercise, mobilisation techniques and taping in patients with subacromial pain syndrome and found insufficient evidence to support for the use of scapular-focused treatment [63, 64].

Similar to the results of this present review, both previous reviews found significant methodological limitations among the available evidence [63, 64]. One review [63] found the literature not to be supportive of different scapular-focused treatment approaches for pain and function; All studies investigated the short-term effects of exercise on pain at 4 to 8 weeks follow-up; whereas the meta analysis of one review found statistical, but not clinically relevant benefit for the use of scapular-focused treatment in the short term function [64]. These previous reviews have limited value for assessing the effectiveness of exercise as the exercises identified focused on scapular position and movement disregarding evidence that resistive exercises are superior [18, 28, 32, 63, 64]. Moreover, these previous reviews lack investigation of proprioceptive exercise protocols which, in the review authors’ opinion, is an important aspect of specific exercise strategy in rehabilitation programs aimed at correction of movement and kinematics.

In contrast to previous reviews, this is the first systematic review specifically examining effectiveness of specific resistive exercises compared with general exercise in the rehabilitation of patients with SIS.

One previous systematic review evaluate the role of exercise in treating rotator cuff impingement and found a strong suggestion that exercise improves symptoms in patients with SIS [34]. This review included exercise programs with components of home-exercises, manual therapy and other modalities [34] and therefore does not provide evidence supporting specific exercises as more effective then general exercise interventions.

Previous recommendations from a consensus statement, which supported the use of scapular rehabilitation protocols within a comprehensive program to potentially improve scapular muscle strength, shoulder symptoms and alter scapular position [17], are not supported by the results of this review.

A small number of studies were found comparing the use of specific resistive exercise with movement-based exercises without external resistance in rehabilitation of SIS and were deemed unfit for inclusion [28, 40, 65–67]. Furthermore, other reviews offer evidence that resistance training of any type is superior to movement based training only [16, 18, 30, 32, 68]. Therefore, it would be difficult to determine effectiveness of specific exercise from these studies. One excluded study tested the effects of a neurocognitive approach compared to traditional therapeutic exercises in patients with SIS [66], and found the neurocognitive approach to result in larger improvement in shoulder function and pain [66]. Interestingly, the traditional therapeutic exercise group engaged in specific resistive exercises which focused on the rotator cuff and scapular stabilizing muscles [66]. These findings could indicate the need for consideration of different styles of intervention for the treatment of SIS patients.

There is currently one completed upcoming RCT that, with reference to the protocol registration at clinicaltrials.gov, could meet inclusion criteria of this review (Elif Turgut NCT02286310) (Appendix 3). The protocol description includes specific exercise therapy for patients with SIS and scapular dyskinesia only, however the results of this upcoming RCT could be considered in future investigations of specific exercise strategy.

Two recent Cochrane systematic reviews evaluating the use of the motor control as a specific exercise strategy among low back [69] and neck pain populations [70]. Both studies suggest specific motor control exercises, aimed at restoring control and coordination of deep stabilizer muscles of the spine, were not superior to more general exercise strategies [69, 70]. Both studies conclude no single exercise strategy is superior to another [69, 70]. In comparison to the results of this present review similar conclusions could be drawn for the use of specific exercises such as scapulothoracic, positioning, proprioceptive and neuromuscular control exercises for the treatment of SIS.

### Strengths and limitations

As suggested by Cochrane, reviewers conducted a wide systematic search strategy with high sensitivity and low precision in order to detect all relevant articles [42]. Despite a comprehensive search process, reviewers collected only six articles suitable for inclusion [53–58]. A limited number of studies of moderate to low quality may have contributed to an over or under estimated effect and thus prevents any clear conclusions. Moreover, there are a relatively low number of participants in this review and it should be noted that high quality RCTs with adequate sample sizes might change the estimate of effect. Wang et al. [56] did not clearly state the inclusion of participants with SIS. It could be argued that this study did not meet inclusion criteria and therefore excluded. One study could not be included in statistical pooling for both pain and function as the presentation of the results limited data extraction [58].

However, excluding this study would not have changed the overall results and conclusion of this review. Limitations such as the unclear definition of SIS combined with the multifactorial nature of SIS could affect the results of this review [6, 7, 60, 61]. To ensure quality the authors underwent several pilot-tests regarding risk of bias assessment and data extraction. Moreover, reviewers conducted this review based on guidelines from credible sources [42, 48, 71].

## Conclusions

### Implications for practice

Despite compelling research on exercise therapy there is insufficient evidence to either support or disprove specific exercises strategies for treatment of patients with SIS.

Due to inconsistencies and lack of high quality among the available evidence, this review is unable to demonstrate whether implementing specific exercises in a rehabilitation program for patients with SIS is relevant for clinical practice. Furthermore, no recommendations about nature of exercises, frequency, dose and intensity can be made.

### Implications for further research

Future studies aiming to investigate specific exercise strategies should aim to minimize potential bias by presenting a clear methodological design for method of randomization, allocation concealment, blinding etc. This includes a clear description of inclusion criteria, criteria for diagnosis of SIS, reproducible and transparent interventions and co-interventions. There is a need for studies with larger sample sizes to ensure adequate power to detect small inter-group differences. Moreover, follow-up schemes over 6 months will aid to determine long-term effects of the intervention.

If future trials find specific exercises to be effective for the treatment of SIS, a dose-response effect is needed to guide clinical practice in regards to frequency, intensity, dosage (sets/repetitions), rest, tempo manipulation, and whether programs should be individualized or standardized.

### Additional files

Additional file 1: Figure S1. Data and forest plot illustrating results of sensitivity analyses specific exercise versus general exercise for short term pain during activity (4–8 weeks) [53, 54, 56]. (TIF 40 kb)

Additional file 2: Figure S2. Data and forest plot illustrating the results of sensitivity analysis for specific exercise versus general exercise for short term function (4–8 weeks) when restricting the analysis to three studies [53, 56, 57]. (TIF 57 kb)

Additional file 3: Figure S3. Data and forest plot illustrating results of sensitivity analysis for specific exercise versus general exercise for short term function (4–8 weeks) when restricting the analysis to three studies [54–56]. (TIF 56 kb)

## Appendix 1

**Table 6:** Database search formulas

ᅟ	ᅟ
PEDro search formula	
#1) impingement	
Body part – upper arm, shoulder or shoulder girdle	
Method – trials	
#2) bursitis	
Body part – upper arm, shoulder or shoulder girdle	
Method – trials	
#3) tend@nitis	
Body part – upper arm, shoulder or shoulder girdle	
Method – trials	
Web of science search formula	
Timespan = All Years, Search language= Auto	
#1) TS = shoulder OR TS = scapula* OR TS = subacromial	
#2) TS = impingement OR TS = bursitis OR TS = Tend*nitis	
#3) TS = rehabilitation OR TS = physiotherapy OR TS = physical therapy OR TS = exercise OR TS = training	
#4) #3 AND #2 AND #1	
#5) #3 AND #2 AND #1 Refined by: DOCUMENT TYPES: (CLINICAL TRIAL)	
PubMed search formula	
#1) “Search ((Shoulder) OR Scapula) OR Subacromial”	
#2) “Search (((Impingement) OR Bursitis) OR Tendinitis) OR Tendonitis”	
#3) “Search ((((Rehabilitation) OR Physiotherapy) OR Physical therapy) OR Exercise) OR Training”	
#4) “Search (((((Shoulder) OR Scapula) OR Subacromial)) AND ((((Impingement) OR Bursitis) OR Tendinitis) OR Tendonitis)) AND (((((Rehabilitation) OR Physiotherapy) OR Physical therapy) OR Exercise) OR Training)”	
#5) “Search (((((Shoulder) OR Scapula) OR Subacromial)) AND ((((Impingement) OR Bursitis) OR Tendinitis) OR Tendonitis)) AND (((((Rehabilitation) OR Physiotherapy) OR Physical therapy) OR Exercise) OR Training) Filters: Clinical Trial”	
Embase Search	
#1) ‘shoulder’/exp OR shoulder OR scapula OR ‘scapula’ OR ‘scapula’/exp OR subacromial	
#2) ‘shoulder impingement syndrome’/exp OR ‘shoulder impingement syndrome’ OR ‘impingement syndrome’/exp OR ‘impingement syndrome’ OR impingement OR brusitis OR ‘tendinitis’/exp OR tendinitis OR ‘tendonitis’/exp OR tendonitis	
#3) ‘rehabilitation’/exp OR rehabilitation OR ‘physiotherapy’/exp OR physiotherapy OR physical AND (‘therapy’/exp OR therapy) OR ‘exercise’/exp OR exercise OR ‘training’/exp OR training	
#4) #1 AND #2 AND #3	
#5) #4 AND (‘clinical trial’/de OR ‘randomized controlled trial’/de)	
#6) #4 AND (‘clinical trial’/exp OR ‘clinical trial’ OR ‘randomized controlled trial’/exp OR ‘randomized controlled trial’)	
#7) #4 AND (‘clinical trial’/exp OR ‘clinical trial’ OR ‘randomized controlled trial’/exp OR ‘randomized controlled trial’) AND ([controlled clinical trial]/lim OR [randomized controlled trial]/lim)	
#8) #4 AND (‘clinical trial’/exp OR ‘clinical trial’ OR ‘randomized controlled trial’/exp OR ‘randomized controlled trial’) AND ([controlled clinical trial]/lim OR [randomized controlled trial]/lim) AND [embase]/lim	
Cochrane Library Search formula	
#1) shoulder or scapula or subacromial	
#2) impingement or bursitis or tendinitis or tendonitis	
#3) rehabilitation or physiotherapy or physical therapy or exercise or training	
#4) #1 and #2 and #3	
#5) MeSH descriptor: [Shoulder] explode all trees	
#6) MeSH descriptor: [Scapula] explode all trees	
#7) MeSH descriptor: [Shoulder Impingement Syndrome] explode all trees	
#8) MeSH descriptor: [Bursitis] explode all trees	
#9) MeSH descriptor: [Tendinopathy] explode all trees	
#10) MeSH descriptor: [Rehabilitation] explode all trees	
#11) MeSH descriptor: [Physical Therapy Modalities] explode all trees	
#12) MeSH descriptor: [Exercise] explode all trees	
#13) MeSH descriptor: [Motor Activity] explode all trees	
#14) MeSH descriptor: [Physical Education and Training] explode all trees	
#15) #5 or #6	
#16) #7 or #8 or #9	
#17) #10 or #11 or #12 or #13 or #14	
#18) #15 and #16 and #17	
#19) #18 or #4	
#20) #18 or #4 in Trials	

## Appendix 2

**Table 7:** Table of excluded studies

Bae 2011 [72]	Intervention not eligible: did not include resistance training in both groups.
Beaudreuil 2011 [28]	Intervention not eligible: did not include resistance training in both groups.
Beaudreuil 2015 [73]	Secondary analysis of Beaudreuil 2011.
Blume 2015 [74]	Intervention not eligible: both groups received the same exercise program with different progression of repetition max.
Boeck 2012 [75]	Intervention not eligible: passive control group.
Celik 2009 [76]	Intervention not eligible: compares exercises performed above and below 90° of levation.
Cheng 2007 [77]	Intervention not eligible: interventions includes many variables and it is therefore not possible to differentiate between which parameter causes a given effect.
Choi 2013 [78]	Intervention not eligible: no information regarding the control group.
De Mey 2012 [79]	Non-RCT.
Dickens 2005 [80]	Intervention not eligible: passive control group.
Ginn 2005 [81]	Intervention not eligible: does not include resistance training in both groups.
Hallgren 2014 [65]	Intervention not eligible: did not include resistance training in both groups.
Holmgren 2012 [36]	Intervention not eligible: did not include resistance training in both groups.
Jung 2012 [82]	Non-RCT.
Krischak 2013 [83]	Population not eligible: includes patients with full-thickness rotator cuff tears.
Kromer 2013 [84]	Intervention not eligible: both groups received the same exercise program. The intervention group additionally received individualized physiotherapy.
Ludewig 2003 [85]	Intervention not eligible: passive control group.
Marzetti 2014 [66]	Intervention not eligible: neurocognitive training vs resistance training.
Morl 2011 [86]	Intervention not eligible: a comparison of exercise apparatuses.
Mozey 2014 [67]	Intervention not eligible: passive control group.
Østerås 2009 [87]	Intervention not eligible: dose response trial focusing on number of repetitions, number of sets and time spent on performing global aerobic exercises.

*Abbreviations*: *RCT* Randomized controlled trial

## Appendix 3

**Table 8:** Table of ongoing studies

Study	Methods	Participants	Intervention	Outcome measures	Start	Contact
Turgut 2016 *Effect Of Exercise Programs On 3-Dimensional Scapular Kinematics, Disability And Pain In Subjects With Shoulder Impingement* NCT02286310	RCT	Ages 18 to 45 years Genders Both Inclusion criteria Subacromial impingement with scapular dyskenisis Exclusion criteria Additional muscular and skeletal or systemic diseases	Experimental (Group A) •Kinetic chain exercises (exercises include kinetic chain segments) Active comparator (Group B) •Traditional exercises (stretching and strengthening exercises)	Primary Outcome Measures •3-Dimensional Scapular Kinematics measured by electromagnetic tracking system •Scapular rotations and orientations in degrees Secondary Outcome Measures •Pain and Disability Status measured by Shoulder Pain and Disability Index (SPADI) •Pain Severity measured by VAS (in millimeters)	Feb 2014	•Elif Turgut, MSc 00903123052525 ext 186, elifcamci@hacettepe.edu.tr •İrem Düzgün, PhD, 00903123052525 ext 186, iremduzgun@yahoo.com •Hacettepe University Recruiting Ankara, Turkey

*Abbreviations*: *RCT* Randomized controlled trial, *VAS* Visual Analog Scale

## Abbreviations

AS: Author Alison R. Shire
CIs: Confidence intervals
DC: Author David Høyrup Christiansen
JO: Author Jesper B. Overby
MD: Author Mathias Bastholm Dahl
QoL: Quality of life
RCTs: Randomized controlled trials
ROM: Range of motion
SDQ: Shoulder Disability Questionnaire
SIS: Subacromial impingement syndrome
SMD: Standardized mean difference
TS: Author Thor A. B. Stæhr
VAS: Visual analogue scale
VNRS: Verbal numeric rating scale
VNS: Visual numeric scale
WORC: Western Ontario Rotator Cuff Questionnaire
