# Supplementary figures and images for: Specific or general exercise strategy for subacromial impingement syndrome–does it matter? A systematic literature review and meta analysis
Source: BMC Musculoskelet Disord. 2017 Apr 17;18:158. doi: 10.1186/s12891-017-1518-0 (PMC5393017; doi:10.1186/s12891-017-1518-0)

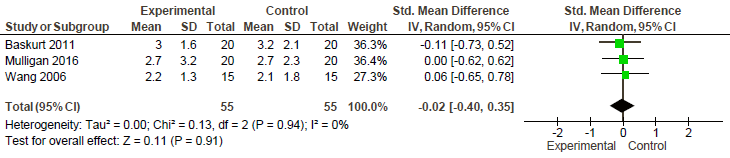

Supplement: Supplementary file 1 — Data and forest plot illustrating results of sensitivity analyses specific exercise versus general exercise for short term pain during activity (4–8 weeks) [53, 54, 56]. (TIF 40 kb) [file 12891_2017_1518_MOESM1_ESM.tif]

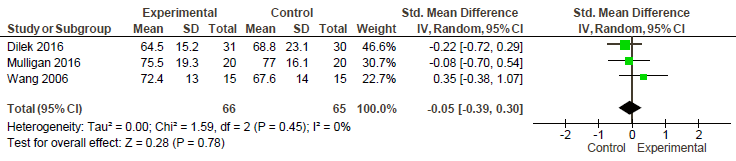

Supplement: Supplementary file 2 — Data and forest plot illustrating the results of sensitivity analysis for specific exercise versus general exercise for short term function (4–8 weeks) when restricting the analysis to three studies [53, 56, 57]. (TIF 57 kb) [file 12891_2017_1518_MOESM2_ESM.tif]

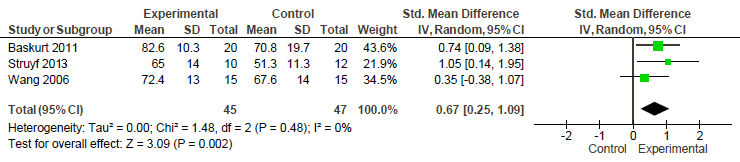

Supplement: Supplementary file 3 — Data and forest plot illustrating results of sensitivity analysis for specific exercise versus general exercise for short term function (4–8 weeks) when restricting the analysis to three studies [54–56]. (TIF 56 kb) [file 12891_2017_1518_MOESM3_ESM.tif]
